# The Plight of Muntaser Ibrahim

**DOI:** 10.1371/journal.pgen.1008100

**Published:** 2019-03-25

**Authors:** Giorgio Sirugo, Scott M. Williams, Sarah A. Tishkoff, Heather J. Cordell, Jonathan Marchini, Gregory S. Barsh, Gregory P. Copenhaver

**Affiliations:** 1 Department of Systems Pharmacology and Translational Therapeutics, University of Pennsylvania Perelman School of Medicine, Philadelphia, PA; 2 Department of Population and Quantitative Health Sciences, Case Western Reserve University, Cleveland, OH, United States of America; 3 Department of Genetics, Perelman School of Medicine, University of Pennsylvania, Philadelphia, PA; 4 Institute of Genetic Medicine, Newcastle University, Newcastle upon Tyne, United Kingdom; 5 Regeneron Genetics Center, Tarrytown, NY; 6 HudsonAlpha Institute for Biotechnology, Huntsville, Alabama, Department of Genetics, Stanford University School of Medicine, Stanford, California, United States of America; 7 Department of Biology and the Integrative Program for Biological and Genome Sciences, University of North Carolina at Chapel Hill, Chapel Hill, North Carolina, United States of America

We wish to bring to the attention of the international scientific community, and in particular the genetics community, the reported detention of Professor Muntaser Ibrahim of the University of Khartoum in Sudan. According to various news sources, Prof. Ibrahim was arrested by Sudanese authorities on the 21^st^ of February [1]. Prof. Ibrahim’s whereabouts are currently unknown and we have deep concerns about his well-being.

Prof. Ibrahim, a leading Sudanese geneticist, is internationally recognized for his work on important medical genetics problems, from infectious diseases to cancer, and for his extensive contributions to our understanding of the genetic complexity of African populations. He has been a leader in high profile EU and UK-funded research initiatives on malaria, tuberculosis and leishmaniasis and a founding member of the African Society of Human Genetics, earning the respect of many international collaborators who have had the privilege of working with him. He is also a dedicated educator and mentor, with profound impact on the careers of Sudanese students who have and continue to train under his supervision.

We admire Professor Ibrahim’s sharp intellect and curiosity, and we believe that he shares a core value with our journal in particular—a passionate belief in free and open discourse. Among those who know him he is well recognized for being respectful with his interlocutors, but also being unflinching in expressing his opinions.

We believe Professor Ibrahim’s contributions to the international scientific community are invaluable and are deeply concerned about the recent reports. We hope that Prof. Ibraham can soon continue his work in making groundbreaking advancements in genetics, educate the next generation of Sudanese scientists, and contribute to building Sudan's international standing as a home for world-class researchers.
